# Assessing Latrine Use in Rural India: A Cross-Sectional Study Comparing Reported Use and Passive Latrine Use Monitors

**DOI:** 10.4269/ajtmh.16-0102

**Published:** 2016-09-07

**Authors:** Antara Sinha, Corey L. Nagel, Evan Thomas, Wolf P. Schmidt, Belen Torondel, Sophie Boisson, Thomas F. Clasen

**Affiliations:** ^1^ Faculty of Infectious and Tropical Diseases, London School of Hygiene and Tropical Medicine, London, United Kingdom; ^2^ School of Public Health, Oregon Health and Science University, Portland, Oregon; ^3^ The Portland State University SWEETLab, Portland, Oregon; ^4^ Department of Environment Health, Rollins School of Public Health, Emory University, Atlanta, Georgia

## Abstract

Although large-scale programs, like India's Total Sanitation Campaign (TSC), have improved latrine coverage in rural settings, evidence suggests that actual use is suboptimal. However, the reliability of methods to assess latrine use is uncertain. We assessed the reliability of reported use, the standard method, by comparing survey-based responses against passive latrine use monitors (PLUMs) through a cross-sectional study among 292 households in 25 villages in rural Odisha, India, which recently received individual household latrines under the TSC. PLUMs were installed for 2 weeks and householders responded to surveys about their latrine use behavior. Reported use was compared with PLUM results using Bland–Altman (BA) plots and concordance statistics. Reported use was higher than corresponding PLUM-recorded events across the range of comparisons. The mean reported “usual” daily events per household (7.09, 95% confidence interval [CI] = 6.51, 7.68) was nearly twice that of the PLUM-recorded daily average (3.62, 95% CI = 3.29, 3.94). There was poor agreement between “usual” daily latrine use and the average daily PLUM-recorded events (ρ_c_ = 0.331, 95% CI = 0.242, 0.427). Moderate agreement (ρ_c_ = 0.598, 95% CI = 0.497, 0.683) was obtained when comparing daily reported use during the previous 48 hours with the average daily PLUM count. Reported latrine use, though already suggesting suboptimal adoption, likely exaggerates the actual level of uptake of latrines constructed under the program. Where reliance on self-reports is used, survey questions should focus on the 48 hours prior to the date of the survey rather than asking about “usual” latrine use behavior.

## Introduction

Improving sanitation is regarded as a key public health measure to reduce infectious diseases.^1^ Latrine use is an important outcome indicator for monitoring the effectiveness of sanitation programs.^2^–^4^ Although large-scale campaigns in India, which prioritize the elimination of open defecation, have succeeded in increasing latrine coverage, actual adoption and use has been suboptimal.^4^–^9^ Poor use may be a partial explanation why recent evaluations of such programs have found that they have not prevented sanitation-related diseases such as diarrhea and soil-transmitted helminth infection.^10^–^12^ Increasing evidence has shown that in settings such as India, an emphasis on latrine access and/or ownership alone, without addressing latrine use, is not likely to yield desired programmatic outcomes, including open defecation free status, health, and other gains from sanitation.^4^,^8^,^9^,^13^,^14^

However, measuring household and individual latrine use is challenging. Direct observation is costly, potentially objectionable, and has shown to cause reactivity.^14^ Spot-checks and latrine use indicators provide only an indication of household use, not individual use.^10^,^11^,^15^,^16^ Some evidence suggests that repeated spot-checks have potential to cause reactivity in longitudinal studies.^17^ Sensor-monitored use based on passive latrine use monitors (PLUMs) or similar devices are useful in assessing the reliability of other methods.^14^ They have identified evidence, for example, of reactivity in using direct observation, previously thought to be the gold standard in assessing latrine use. However, existing sensors are not practical for large-scale latrine use assessment.

Self-reported measures, such as maintaining a diary^18^,^19^ or responding to surveys,^5^,^20^,^21^ are the most common method to measure behavior in water, sanitation, and hygiene interventions. The Joint Monitoring Program for Water and Sanitation (JMP), which currently monitors progress toward international water and sanitation targets, recommends that national surveys ask, “What kind of toilet facility do members of your household usually use?”^22^ In India, the 69th round of the National Sample Survey included a section on “latrine,” which among other items, asked “whether all household members of categories specified are using the latrine” (yes, no, not applicable). The categories were “male of age below 15 years,” “male of age 15 years and above,” “female of age below 15 years,” and “female of age 15 years and above.”^9^ Some studies have used the self-report method as a complementary approach in conjunction with other approaches, including technology-based measures, such as electronic soap loggers, and latrine inspections or spot-checks.^10^,^23^

However, evidence suggests that study subjects tend to over-report desirable behavior in response to survey questions.^3^,^10^,^24^–^27^ Repeated interviews or completing a diary and ensuring that recordings are not missed may be burdensome to investigators and subjects, leading to fatigue and thereby reducing reliability.^28^ Further, household-based surveys that are often used to elicit such information tend to be time consuming and expensive.^3^

In the context of a large-scale trial (the “Sanitation Trial”) to assess the impact of improved sanitation in rural India, we undertook a few approaches to assessing latrine use.^11^,^14^ In this article, we report on various approaches to assessing latrine use based on self-reports at the household and individual level, and compare the results with PLUMs mounted inside the latrine.

## Materials and Methods

### Study context.

The study was conducted among 25 villages in rural Puri, a coastal district of Odisha, India, which comprised the intervention arm of a randomized, controlled trial (the “Sanitation Trial”) to assess the health impact of rural sanitation under the Indian Total Sanitation Campaign (TSC).^11^,^29^ Findings from a baseline survey revealed that approximately 10% of households among the intervention villages had access to a latrine.^29^ Between January 2010 and March 2011, WaterAid and its implementing partners conducted community mobilization and constructed household pour-flush latrines among eligible “below the poverty line” households.^30^

### Village and household selection.

This latrine use study was conducted among 25 of the 50 villages comprising the intervention arm in the Sanitation Trial. Villages were eligible if they had at least one household that was included in the Sanitation Trial surveillance (had a child under 4 years and/or a pregnant woman at baseline) with a functional latrine as a result of the intervention (a surrounding wall/ enclosure, a door/closure over the entrance for privacy, an unbroken toilet pan, a functional pan-pit connection, and the presence of a covered pit). A total of 46 villages were found to be eligible from which 25 were randomly selected for the latrine use study using block-level stratification and a computer-generated sequence. All surveillance households in the selected villages were eligible to participate in the latrine use study provided they had functional latrines. Eligible households were enrolled if they consented to participate in the study.

### Surveys to assess latrine use.

In this article, we compare various approaches to assessing reported use both at the household and individual level with results from PLUMs. Both these methods were pilot tested extensively in the field in 2011 and 2012 before arriving at the final versions that were ultimately used in this study. Reported latrine use was assessed by trained enumerators using a survey-based instrument translated into the local language. The survey included questions on whether the household has access to a latrine, whether they owned a latrine, whether any members of the household have “ever use(d)” the latrine since it was constructed, and whether any members of the household used any other latrine in the village. It then went on to capture latrine use data for each member of a given household, thereby enabling an assessment both at individual and household levels. This study used data obtained through three main survey questions to enable a valid comparison with concurrently obtained PLUM-recorded data for the given household: “usual” or average daily latrine use; latrine use “yesterday” (or the last day of the observation period); and latrine use the “day before yesterday” (or the second last day of the observation period). The fourth comparative category, which was latrine use in the last 48 hours of observation, was a derived measure that was a summation of latrine use “yesterday” and the “day before yesterday.” These categories were selected to enable a comparative assessment of the two measures in the context of an extended perspective of use (“usual” latrine use behavior), and a more time-bound perspective of use (latrine use behavior for “yesterday,” the “day before yesterday,” or the last 48 hours).

### Passive latrine use monitor.

The PLUM represents the fourth generation of a device described elsewhere.^14^ The device was developed by Portland State University in the United States (www.pdx.edu/sweetlab). Mounted in a latrine, the battery powered device employs a passive infrared (PIR) motion sensor to detect the presence or absence of warm-body movement within its viewing range. An algorithm developed and validated based on a previous generation of the device is used to interpret the raw data and generate estimates of likely “defecation events.” The algorithm distinguished likely nondefecation events as those characterized by dense motion-based triggering in the PLUM under 30 seconds with no similar triggers within 10 minutes before or after.^14^

### Household follow-up procedure.

Based on data from a sample of 30 households where the PLUM had been installed as part of a pilot study in 2011–2012, we determined a within household correlation of mean PLUM-recorded events over an average of 42 observation days per household was high (intraclass correlation coefficient = 0.38) and that repeat measurements of more than 14 days in a household per round would yield little gain in study power. We therefore selected a 2-week follow-up period. Some of the results from this survey will be reported in another paper.

PLUMs were installed in eligible household latrines for a 16-day period. Days 1 and 16 that corresponded to installation and removal dates were dropped to reduce errors. Data from the intervening 14-day period were used. If a household owned more than one latrine, PLUM devices were installed in each of those latrines. Since we found that cellular coverage was poor in the study area, we installed majority of the PLUMs in a local logging mode to ensure that data were recorded and safely stored. These data were later uploaded to a MySQL server for analysis.

Data on reported latrine use were collected for each individual household member in a given household. Questions on reported use were administered to all household members that were present and were able to comprehend and respond to queries. In the event that a household member was not present or was unable to answer the questions, the consenting female head of household or the eldest daughter-in-law was considered the primary household respondent, and provided information on latrine use for those household members. The reported latrine use survey was conducted at the start of the monitoring period (on the same day that the PLUM was installed in the household) except for two questions on the frequency of latrine use “yesterday” and the “day before yesterday,” which were administered at the end of the monitoring period (on the day that the PLUM was retrieved from the household). The frequency of latrine use was recorded only for those members currently living in the household, and visitors, if any, to ensure a more accurate estimate of the total number of household members at the time of data collection.

With regard to reported latrine use for “yesterday” and the “day before yesterday,” each reported 24-hour period was divided into four segments (sunrise/morning; pre-noon/afternoon; evening/sunset; night), and reported events were queried during each segment for each household member to aid more accurate recall. As with the more general question regarding overall use, all household members who were present were asked to report their use and the primary household respondent was asked about latrine use of household members who were unavailable and/or unable to respond. Additionally, the respondent was asked to recall if they had visitors/non-household members on that specific day who may have used the latrine. If they did, similar latrine use data for the visitor(s) were recorded with a distinct coding for the visitor(s). This was done to increase accuracy of reported use by all individuals who may have used the latrine in the specified time.

Additionally, latrine spot-checks were conducted by trained observers as an additional means to assess latrine use in all households on the day that the PLUM was removed, that is, day 16. The four latrine spot-check indicators that were considered were 1) evidence that latrine is used as storage (where storage indicated non-use); 2) leaves/dirt in toilet pan (where the presence of leaves/ dirt indicated non-use); 3) water container in/near latrine for washing (where the presence of a water container indicated use); and 4) slippers outside or inside the latrine (where the presence of slippers indicated use).

Table 1 highlights the questions and methods used for assessing reported latrine use to enable a comparison with a corresponding PLUM-recorded measure for four categories. The estimation approaches used for both measures are also included.

### Data analysis.

The survey data were entered using EPIData 3.1 (EpiData Association, Odense, Denmark). Data were processed and analyzed using STATA 12 (StataCorp, College Station, TX)^31^ and R (Version 3.1.2; R Foundation for Statistical Computing, Vienna, Austria).^32^ Agreement between PLUM-recorded latrine use and reported latrine use was assessed for both the usual latrine use item and the items regarding use in the prior 48 hours as presented in Table 1. The comparison of average reported daily use on days 13 and 14 with the average daily PLUM-recorded count across the total monitoring period was to determine whether the more targeted recall items had better agreement with overall usage patterns than did the more general “usual use” item.

Bland–Altman (BA) plots were constructed to assess agreement between reported latrine use and PLUM-derived count for each of the comparisons listed in Table 1. Because the simple BA method assumes that both the mean and standard deviation (SD) of the differences between methods are constant across the range of measurement, we used the approach suggested by Bland and Altman to assess these assumptions and constructed adjusted plots that accounted for non-constant bias and/or variance.^33^ The steps in this approach were as follows:

1. Given *R*_*i*_ = reported use in household *i* and *P*_*i*_ = PLUM-derived use in household *i*, the difference between reported use and PLUM-derived use was calculated as *D*_*i*_ = *R*_*i*_ − *P*_*i*_, and the average of reported use and PLUM-derived use was calculated as *Λv*_*i*_ = (*R*_*i*_ + *P*_*i*_)/2
2. The mean bias between methods was modeled using linear regression as *D*_*i*_ = *a* + *b*(*Λv*_*i*_). Non-constant bias is indicated by *b* > 0
3. The absolute residuals from the model specified in step 2 were regressed on the average (*Λv*) of the methods, *R*_*i*_ = α + β(Λ*v*_*i*_). Non-constant variance (heteroscedasticity) is indicated by β > 0
4. As the absolute residuals from step 3 follow a half-normal distribution, the relationship of the SD of the differences to the average of the measurements is given as 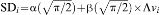. Therefore, the 95% limits of agreement for the difference between the two methods given their average were calculated as *D*_*i*_ ± 2(SD_*i*_)_._

The mean difference between methods and 95% limits of agreement were plotted against the average of the methods per conventional BA plot format.

Next, to model the direct relationship between reported use and PLUM count for each category of comparison, symmetric prediction equations with corresponding 95% prediction intervals were derived from the results of the BA analysis.^34^ Using the parameter estimates from the previous equations, the predicted PLUM-derived count for a given value of reported use was calculated as:

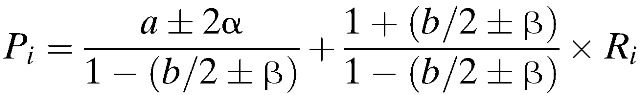


and predicted reported use for a given PLUM-derived count was calculated as:

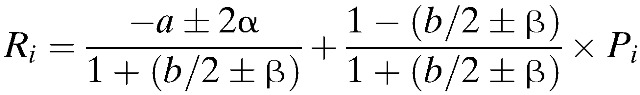


Finally, the concordance correlation coefficient (CCC) was calculated for each pair of measures using the “concord” package.^35^ The CCC is a standardized measure of the variation of the linear relationship between two methods from the 45° line through the origin (the line of perfect agreement). A CCC value of 1 indicates perfect concordance between the measures, whereas a value of 0 indicates a complete lack of concordance. The CCC is a more appropriate method for assessing agreement than the often used Pearson correlation coefficient as the CCC measures both precision, the deviations of the observations from the line of best fit, and accuracy, the distance of the fit line from the line of perfect agreement.^36^ We generated bootstrap 95% confidence intervals (CIs) for the CCC (bias corrected accelerated based on 2,000 bootstrap replicates). To assess for significant differences in the concordance of reported use with PLUM events across the comparison categories, we generated bootstrap 95% CIs (2,000 replicates) of the difference between CCCs using the approach described by Crawford and others.^36^

With reference to the four latrine spot-check indicators, we conducted an additional series of analyses to assess whether incorporating information from the four selected latrine spot-check items reduced the observed bias in reported latrine use relative to the PLUM-recorded events. Specifically, if household members reported latrine use but the latrine spot-check item indicated non-use, the reported use for that household was given a value of 0. In households with multiple latrines, the nonuse condition needed to be met in all the latrines for the given household. The CCC and the limits of agreement from the BA plot were recalculated with the adjusted values and compared with the unadjusted reported values. This comparison was conducted independently for each of the spot-check items as well as for the combined presence of any of the indicators.

### Ethics.

The latrine use assessment research was a sub-study of the Sanitation Trial and was granted ethics approval by the Ethics Committee of the London School of Hygiene and Tropical Medicine (Approval #5561, as amended), and by the Institutional Ethics Committee of the Xavier University, Bhubaneswar (Approval #310510, as amended). The Sanitation Trial was registered with ClinicalTrials.gov (Registration No. NCT01214785). Participants in the research were provided full details of the study prior to seeking informed, written consent from the male/female head of the household. In addition, Village Water and Sanitation Committee members were also consulted prior to initiation of the study. Measures were taken to ensure confidentiality for all participants.

## Results

**Figure 1.**
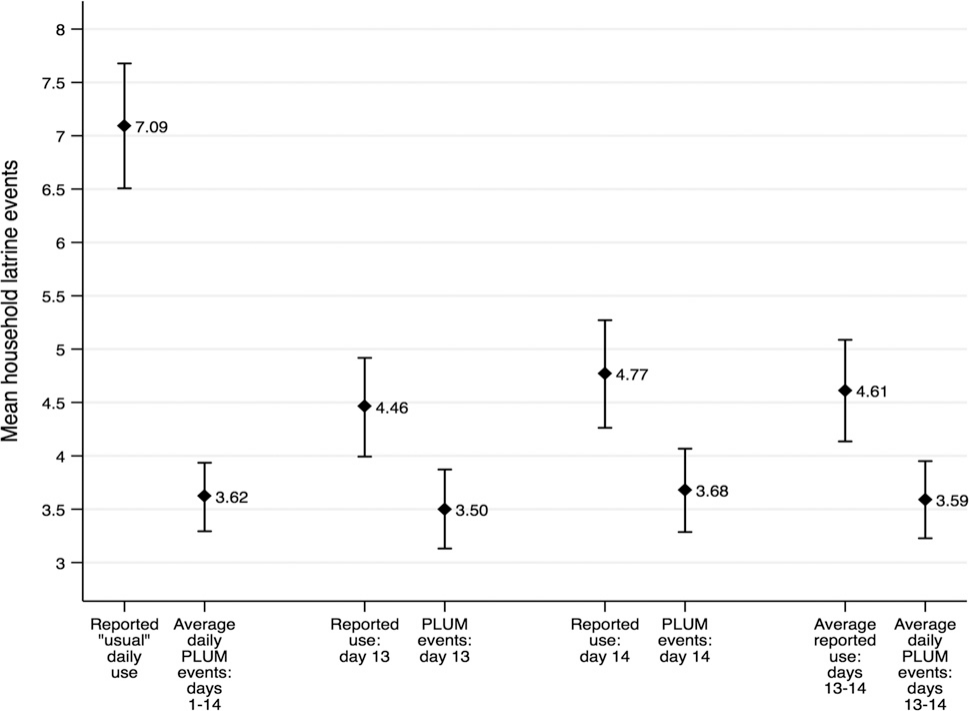
Mean latrine events and 95% confidence interval for households (*N* = 292) for reported latrine use and corresponding PLUM-recorded latrine use for varying time. The average reported use events are consistently greater than the corresponding PLUM-recorded latrine events for all four comparison categories.

We obtained results on latrine use from 292 households. With 14 days of surveillance data per household, the study includes a total of 4,088 days of household-level latrine use data for 2,035 individuals, including 31 visitors. The average household size was 6.74 (SD = 3.02) with a range from 2 to 29 members per household. Comparison of reported latrine use and PLUM-recorded latrine events revealed that, on average, the reported use measures were higher than the corresponding PLUM-recorded latrine events across the range of comparisons (Figure 1

). The mean reported “usual” daily events (7.09, 95% CI = 6.51, 7.68) was nearly twice as high as that of the PLUM-recorded daily average (3.62, 95% CI = 3.29, 3.94). Reported use on days 13 and 14 were also higher than their corresponding PLUM-recorded latrine events, but that difference was markedly less. The average PLUM-recorded latrine events were similar for the 14-day observation period (3.62, 95% CI = 3.29, 3.94) and for the last 48 hours (3.59, 95% CI = 3.23, 3.95). It may therefore be reasonable to compare the PLUM-recorded daily average for the 14-day observation period with average reported use for the prior 48 hours in the fourth category. For the “usual” or average daily reported use measure, the proportion of self-report to report was 25.3% self-report, 74.7% reported. For the 48-hour recall measure, it was 24.0% self-report and 76.0% reported.

### Assessing agreement using BA plots.

**Figure 2.**
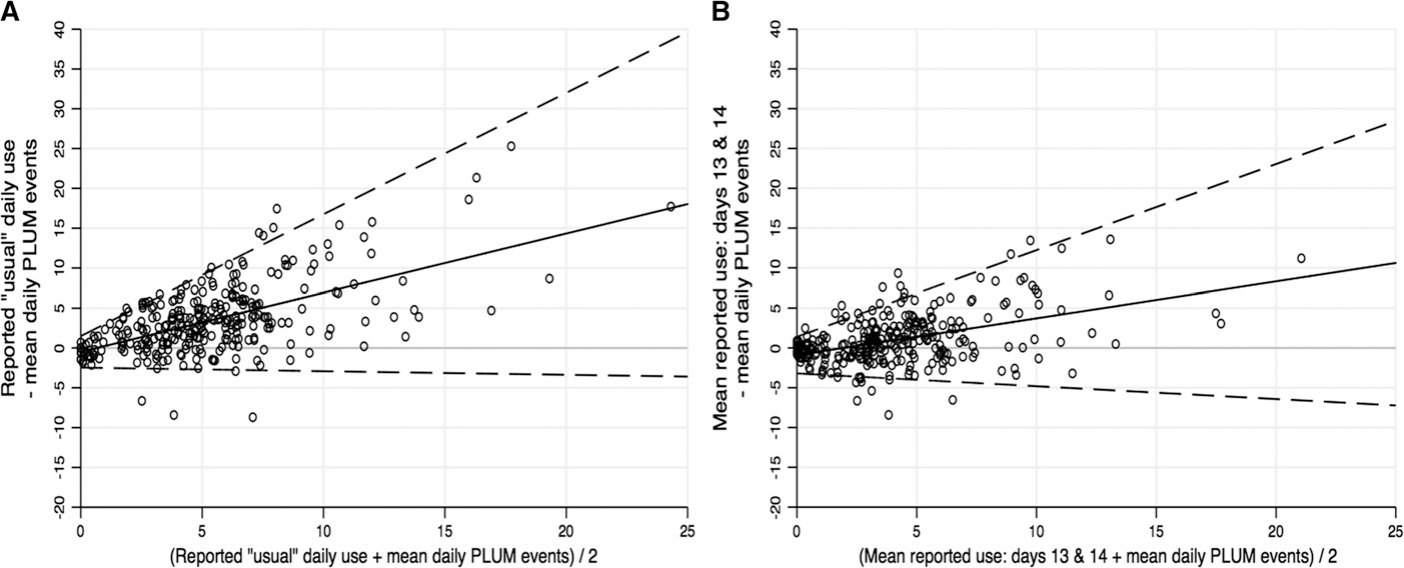
Bland–Altman plots comparing (**A**) reported “usual” daily latrine use with average daily PLUM-recorded latrine events, (**B**) average of reported use on days 13 and 14 with average daily PLUM-recorded events during the total observation period. The mean difference between methods (bias) is shown by the solid line and the dashed lines show the 95% limits of agreement, which is the interval expected to contain 95% of the differences between methods. For each comparison, both the mean difference and the variance between methods are observed to increase as the magnitude of the measurement increases.

In each of the four categories, the results of regressing the difference between PLUM events and reported use on their average indicated nonconstant bias between the methods. Similarly, there was a significant positive relationship between the absolute residuals from the previous step and the average of the methods in each category, indicating nonconstant variance between PLUM derived-use and reported use. Figure 2

presents the BA plot of the difference between the two methods against their average for the two main comparison categories—reported “usual” daily latrine use with average daily PLUM-recorded latrine events and the average of reported use on days 13 and 14 with average daily PLUM-recorded events during the total observation period. The BA plots comparing reported use on day 13 with PLUM-recorded events on day 13, and reported use on day 14 with PLUM-recorded events on day 14 are included in the supplementary information material (Supplemental Figure 1A and B).

**Figure 3.**
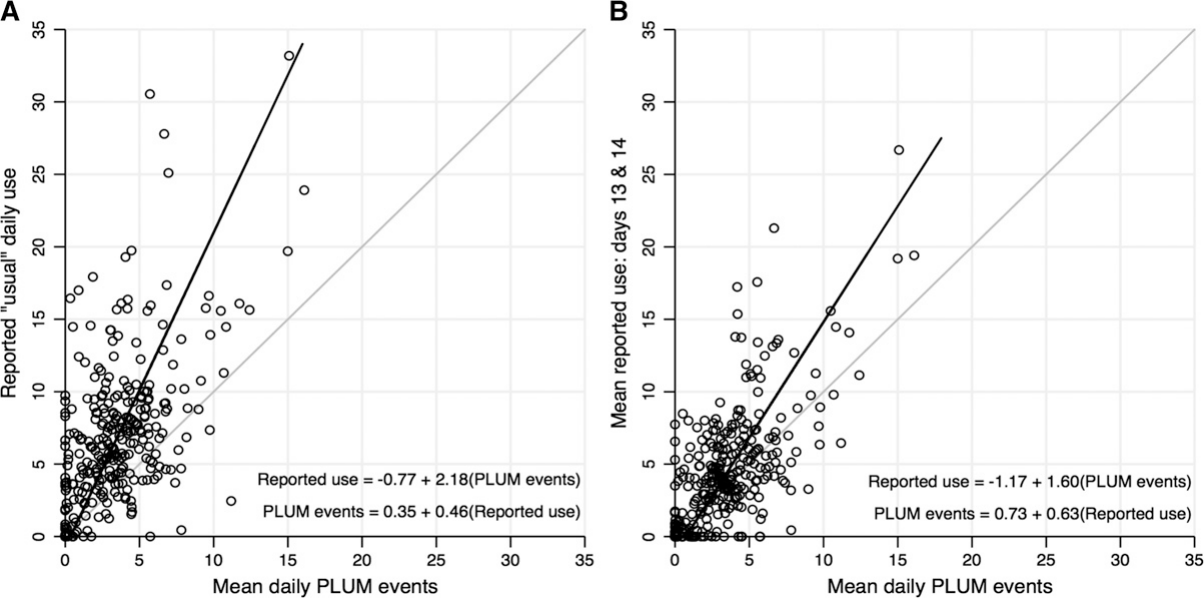
Scatterplots of (**A**) reported “usual” daily latrine use and average daily PLUM-recorded latrine events, (**B**) average of reported use on days 13 and 14 and average daily PLUM-recorded events during the total observation period. Symmetric prediction equations allowing for direct conversion between the methods are derived from the Bland–Altman analysis. The predicted value of one method (e.g., reported use) given the other (e.g., PLUM events) is displayed by the solid line. The shaded 45° line at the origin is the line of equality, indicating perfect agreement between the methods.

Across the comparisons, there was a pattern of upward bias in the difference between reported use and PLUM events, indicating that, on average, households over-reported latrine use relative to the PLUM-recorded events during the observation period. The magnitude of this difference was greatest between reported “usual” latrine use and the average household PLUM-recorded events (Figure 2A). The equations derived from the BA analysis indicate that reported “usual” daily use was, on average, 118% higher than the average number of PLUM events recorded in the household (Figure 3A

). Notably, when respondents were asked about use in the households on days 13 (day before yesterday) and 14 (yesterday), the bias between reported use and PLUM events on the corresponding day was reduced (Supplemental Figures 1, 2A and B). Across the comparison categories, the 95% limits of agreement were fairly wide.

Given the reduction in bias observed between the reported measures of daily latrine use in the prior 48 hours with the PLUM-recorded latrine events for those days, we averaged the reported use “yesterday” and the “day before yesterday” within each household and compared that to their average daily PLUM-recorded events across the 2-week observation period. As displayed in the BA plot (Figure 2B), the average bias between the 48-hour recall measure and the average daily PLUM-recorded events was less than that with the measure of reported “usual” latrine use. The predicted frequency of latrine use with the 48-hour recall measure was 60% higher than the average daily PLUM-recorded events over the 2-week study period (Figure 3B).

### Concordance correlation coefficient.

The results obtained from calculation of the concordance correlation coefficient were also found to be aligned with the results of the BA analysis. There was poor concordance between reported “usual” daily latrine use and the average daily PLUM-recorded events (ρ_c_ = 0.331, 95% CI = 0.242, 0.427). The concordance between reported use on day 13/the “day before yesterday” and the corresponding day's count of PLUM events was 0.467 (95% CI = 0.334, 0.560). We found that agreement further improved between reported use for day 14/“yesterday” and the PLUM count for the same 24-hour period (ρ_c_ = 0.581, 95% CI = 0.476, 0.688). Finally, the CCC (ρ_c_ = 0.598, 95% CI = 0.497, 0.683) for reported use in the last 48 hours and PLUM-recorded use over 14 days indicated an improvement in precision and a moderate agreement between the two measures. The concordance between the 48-hour recall measure and the average PLUM-recorded events was significantly higher than that between the “usual” latrine use measure and the average PLUM count (95% CI of the difference: 0.21, 0.32, *P* < 0.05).

The use of the four latrine spot-check indicators to adjust reported latrine use in households where visual inspection suggested that the latrine was not being used resulted in negligible improvements in both the CCC and the limits of agreement from the BA plot (data not shown).

## Discussion

We found that average reported latrine use was consistently higher than average PLUM-recorded latrine use over all four categories of comparison considered in this study. This is consistent with previous literature, which indicates that relying on reported sanitation behavior via surveys may be subject to courtesy and recall bias and may influence the behavior being monitored.^3^,^27^,^28^ Additionally, the magnitude of this observed bias was dependent on the category or type of reported latrine use measure. The largest bias was observed with the most general item that queried “usual” number of times per day that a participant used the latrine. This may be because of higher recall bias in instances when recall is not bound by a defined time, such as when responding to “usual” latrine use practices. Our results indicate that the bias was reduced with the measures that compared reported latrine use in the prior 48 hours to corresponding PLUM-recorded use during that time. A plausible explanation for this may be that when queried about latrine use behavior in the prior 48 hours, householders were asked more precise questions with references to clearly defined time. For example, they were asked to respond to each day separately, that is, reported use for yesterday and for the before yesterday. Further, each day was broken into four segments corresponding to sunrise/morning; pre-noon/afternoon; evening/sunset; night/pre-sunrise hours, to facilitate greater accuracy of responses to these time-bound segments. Additionally, visual aids were used to facilitate the understanding of illiterate participants in the study sample. This design may have helped to reduce over-reporting for the relevant periods.

Among the categories of reported latrine use measures, agreement between reported use and PLUM-recorded events was fairly low. Although agreement between average reported use of latrine(s) over the prior 48 hours and average daily PLUM-recorded events for the 2-week period was higher than all the previous measures, it was still less than 0.6 (ρ_c_ = 0.598, 95% CI = 0.497, 0.683). However, it is noteworthy that reported daily use during the previous 2 days was a significantly less biased and more precise measure of average daily PLUM-recorded latrine use across the entire study period than was the more general question about “usual” latrine use. This has implications for how reported use measures are developed and administered in future studies.

It is important to note that the PLUM has not yet been established as the “gold standard” for evaluating other methods for latrine use assessment. There are limitations associated with the PLUM algorithm, which may warrant further evaluation in future studies. Although the algorithm has been refined based on previous research and subsequent small scale testing, it is limited in its ability to disambiguate latrine events that occur within short inter-arrival times.^14^ Consequently, there may be an underestimation of discrete events during peak use times, although it is unlikely that this alone could account for the magnitude of the difference observed in this study. There is also a possibility of behavioral reactivity or reporting bias induced by the presence of the PLUM in the latrine, which may influence the estimation of the bias between reported and PLUM-recorded use. Moreover, the device does not definitively distinguish between the nature of latrine activities, such as the disposal of child feces, which is critical to ensuring sanitary gains,^37^,^38^ urination, or menstrual hygiene. While estimates of average use per person per day may be derived from the aggregated household-level PLUM-recorded events, unlike the (self-) reported use measure, it does not permit a distinction between users and nonusers in a given household or help in profiling those refractory members, so that they may be targeted through further interventions.

Other limitations of this study include a relatively small sample size because of the limited number of PLUMs that were available, each of which had to be installed for a period of 2 weeks per latrine. In households that had multiple latrines, one PLUM was installed per latrine. The study was limited to only those households that were part of the intervention arm of the Sanitation Trial. Therefore, any generalizations made to the larger population would need to be done with caution. Although data were gathered synchronously by the reported use survey and the PLUM for the latrine use measures for “yesterday” and the “day before yesterday,” it was not possible to do so for the “average daily use” category. It was assumed that “usual” daily reported latrine use might be comparable with PLUM-recorded latrine use counts obtained over the 2-week monitoring period. The discrepancy we observed between respondent recall of visitors in the prior 2 days, when households accounted for visitors, compared with that for the first 12 days of monitoring, when respondent recall was poor, suggests the presence of recall bias in our “usual” daily reported use data. In such cases, relying exclusively on the measure of reported use may result in an underestimation of latrine use. There may also be a possibility of courtesy bias in respondent reporting given that the survey focused on sanitation.

Despite these limitations, this study furthers research on the methods for assessing latrine use in low-income settings and adds to a growing body of evidence on the feasibility of instrumented monitoring of sanitation behavior at the household level.^14^,^39^ This is particularly significant in the context of latrine use assessment since such alternatives are likely to offer a viable low-cost, objective, non-invasive and medium to long-term perspective of use. Based on our study data, we may also conclude that while all the categories of reported use are biased compared with the PLUM-based measurement, the aggregated 48-hour recall of individual latrine use in households is the least biased and provides a more accurate measure of overall household latrine use than does the general recall. This measure of reported use may therefore be a useful approach to assess household-level latrine use behavior when sensor-based monitoring alternatives are infeasible.

## Supplementary Material

Supplemental Figures.

## Figures and Tables

**Table 1:** Questions and methods used for assessing reported use of latrines and the corresponding PLUM-recorded estimation approaches for four comparison categories

Parameter	Survey question (asked in Oriya)	Approach to estimate reported use	Corresponding PLUM-recorded estimation
“Usual” or average daily reported latrine use	Among your family members who use the latrine, can you please tell me how many times in the day they usually use the latrine?	Average daily reported use for a given household: sum of “usual” reported latrine use per day for all latrine using household members	Average daily PLUM-recorded use for a given household: sum of PLUM-recorded defecation events over 14 days/14 days (for households without any reported visitors) or sum of PLUM-recorded defecation events over 12 days/12 days (for households reporting visitors on days 13 and 14)
Reported latrine use for “yesterday” (day 14)	For each member of your household, please tell us which members used the latrine for defecation “yesterday” and the approximate time of day they used it. If they used the latrine, tell us the number of times they used it (based on four dis-aggregated parts of the day. Visual aids depicting the parts of the day and household members used to facilitate recall).	Sum of reported latrine events across all parts of the day for all household members for “yesterday” in a given household	Sum of PLUM-recorded defecation events for the same day in the same household
Reported latrine use for the “day-before yesterday” (day 13)	For each member of your household, please tell us which members used the latrine for defecation the “day before yesterday” and the approximate time of day they used it. If they used the latrine, tell us the number of times they used it (based on four dis-aggregated parts of the day. Visual aids depicting the parts of the day and household members used to facilitate recall)	Sum of reported latrine events across all parts of the day for all household members for the “day before yesterday” in a given household	Sum of PLUM-recorded defecation events for the same day in the same household
Reported latrine use −48-hour recall	No separate question asked	Sum of total reported use for “yesterday” and the “day before yesterday”/2: to estimate average reported use based on prior 48-hour recall for a given household	Average daily PLUM-recorded use for a given household based on the 14-day (or 12-day) monitoring period
