# Supplementary material for: Assessing Latrine Use in Rural India: A Cross-Sectional Study Comparing Reported Use and Passive Latrine Use Monitors
Source: Am J Trop Med Hyg. 2016 Sep 7;95(3):720–7. doi: 10.4269/ajtmh.16-0102 (PMC5014284; doi:10.4269/ajtmh.16-0102)
Supplement: Supplementary file 1 [file SD9.pdf]

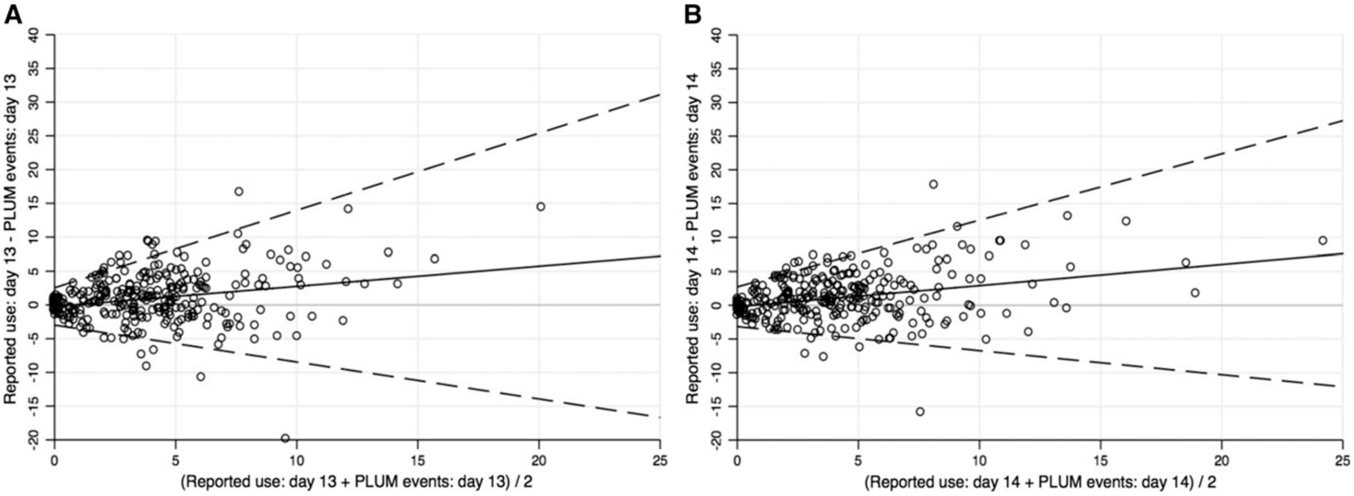

Supplemental Figure 1 Bland–Altman plots comparing (**A**) reported use on day 13 with PLUM-recorded events on day 13, (**B**) reported use on day 14 with PLUM-recorded events on day 14. The mean difference between methods (bias) is shown by the solid line and the dashed lines show the 95% limits of agreement, which is the interval expected to contain 95% of the differences between methods. For each comparison, both the mean difference and the variance between methods are observed to increase as the magnitude of the measurement increases.

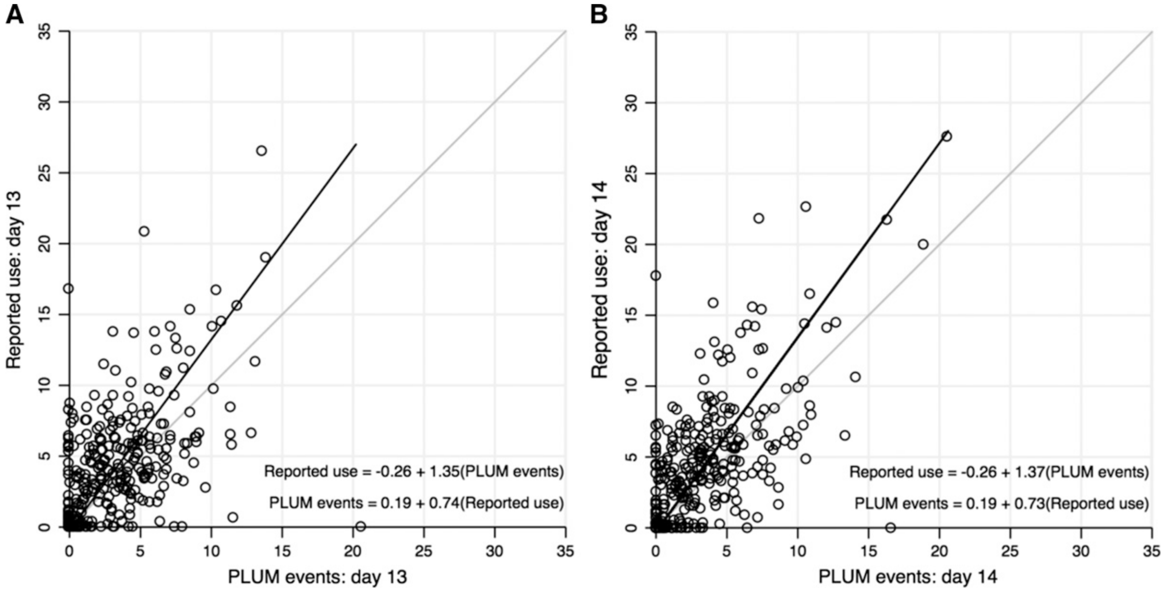

Supplemental Figure 2 Scatterplots of (**A**) reported use on day 13 and PLUM-recorded events on day 13, (**B**) reported use on day 14 and PLUM-recorded events on day 14. Symmetric prediction equations allowing for direct conversion between the methods are derived from the Bland–Altman analysis. The predicted value of one method (e.g., reported use) given the other (e.g., PLUM events) is displayed by the solid line. The shaded 45° line at the origin is the line of equality, indicating perfect agreement between the methods. In Figure 2A, reported use was, on average, 35% higher than recorded PLUM events on day 13 and in Figure 2B, it was 37% higher than recorded PLUM events on day 14.
